# Evaluating whether the proportional odds models to analyse ordinal outcomes in COVID-19 clinical trials is providing clinically interpretable treatment effects: A systematic review

**DOI:** 10.1177/17407745231211272

**Published:** 2023-11-20

**Authors:** Masuma Uddin, Nasir Z Bashir, Brennan C Kahan

**Affiliations:** 1 MRC Clinical Trials Unit at UCL, London, UK; 2 School of Dentistry, University of Leeds, Leeds, UK; 3 MRC Integrative Epidemiology Unit, University of Bristol, Bristol, UK; 4 School of Mathematics and Statistics, The University of Sheffield, Sheffield, UK

**Keywords:** Ordinal outcome, proportional odds model, randomised trial, COVID-19, estimand

## Abstract

**Background:**

After an initial recommendation from the World Health Organisation, trials of patients hospitalised with COVID-19 often include an ordinal clinical status outcome, which comprises a series of ordered categorical variables, typically ranging from ‘Alive and discharged from hospital’ to ‘Dead’. These ordinal outcomes are often analysed using a proportional odds model, which provides a common odds ratio as an overall measure of effect, which is generally interpreted as the odds ratio for being in a higher category. The common odds ratio relies on the assumption of proportional odds, which implies an identical odds ratio across all ordinal categories; however, there is generally no statistical or biological basis for which this assumption should hold; and when violated, the common odds ratio may be a biased representation of the odds ratios for particular categories within the ordinal outcome. In this study, we aimed to evaluate to what extent the common odds ratio in published COVID-19 trials differed to simple binary odds ratios for clinically important outcomes.

**Methods:**

We conducted a systematic review of randomised trials evaluating interventions for patients hospitalised with COVID-19, which used a proportional odds model to analyse an ordinal clinical status outcome, published between January 2020 and May 2021. We assessed agreement between the common odds ratio and the odds ratio from a standard logistic regression model for three clinically important binary outcomes: ‘Alive’, ‘Alive without mechanical ventilation’, and ‘Alive and discharged from hospital’.

**Results:**

Sixteen randomised clinical trials, comprising 38 individual comparisons, were included in this study; of these, only 6 trials (38%) formally assessed the proportional odds assumption. The common odds ratio differed by more than 25% compared to the binary odds ratios in 55% of comparisons for the outcome ‘Alive’, 37% for ‘Alive without mechanical ventilation’, and 24% for ‘Alive and discharged from hospital’. In addition, the common odds ratio systematically underestimated the odds ratio for the outcome ‘Alive’ by –16.8% (95% confidence interval: –28.7% to –2.9%, *p* = 0.02), though differences for the other outcomes were smaller and not statistically significant (–8.4% for ‘Alive without mechanical ventilation’ and 3.6% for ‘Alive and discharged from hospital’). The common odds ratio was statistically significant for 18% of comparisons, while the binary odds ratio was significant in 5%, 16%, and 3% of comparisons for the outcomes ‘Alive’, ‘Alive without mechanical ventilation’, and ‘Alive and discharged from hospital’, respectively.

**Conclusion:**

The common odds ratio from proportional odds models often differs substantially to odds ratios from clinically important binary outcomes, and similar to composite outcomes, a beneficial common OR from a proportional odds model does not necessarily indicate a beneficial effect on the most important categories within the ordinal outcome.

## Background

Randomised trials of patients hospitalised with COVID-19 often include ordinal ‘clinical status’ outcomes,^1,2^ which measure a patient’s clinical status at a given time point using a scale of ordered clinical outcome categories. For instance, a commonly used ordinal scale ranges between ‘1 = Death’, ‘2 = Hospitalised on mechanical ventilation’, up to ‘7 = Not hospitalised’. Ordinal outcomes are sometimes preferred to simple binary outcomes (such as ‘Alive vs dead’), as they incorporate additional information on patients’ health status, for instance, by distinguishing between patients who are alive and discharged from hospital versus those who are still in hospital on mechanical ventilation, which are two very different health states. This can enhance both clinical relevance and statistical efficiency.^3,4^

Ordinal outcomes are frequently analysed using a proportional odds model, which provides a single overall ‘common’ odds ratio (OR).^5,6^ This analysis relies on the proportional odds assumption, which requires there to be an identical OR across each category of the ordinal scale. For instance, for the three-level ordinal outcome in hypothetical trial in Table 1 (1 = Dead, 2 = Alive with mechanical ventilation, 3 = Alive without mechanical ventilation), the proportional odds assumption requires that the odds ratio for categories 1 versus 2–3 be the same as for categories 1–2 versus 3. Then, the common OR represents the OR for being in a higher category of the ordinal scale on intervention compared to control.^7^ For example, consider the hypothetical data in the first three columns of Table 1; the common OR is 1.5, which can be interpreted as the intervention’s effect on each level of the ordinal scale, that is, the intervention increases both the odds of being alive versus dead by 50% and also the odds of being alive without mechanical ventilation versus on mechanical ventilation or dead by 50%. The use of the common OR when the proportional odds assumption holds can confer substantial sample size savings;^8^ in order to detect a common OR of 1.5, 764 patients are required, compared to the 2526 patients required to detect an OR of 1.5 for the binary outcome of ‘Alive vs dead’.

**Table 1.** Sample size requirements for an ordinal outcome versus binary outcome in a hypothetical trial, both when the proportion odds assumption is and is not true.

	Proportional odds assumption true		Proportional odds assumption not true	
	Control (% events)	Intervention (% events)	Control (% events)	Intervention (% events)
Category of ordinal scale				
1. Dead	10%	6.9%	10%	2.5%
2. Alive with mechanical ventilation	35%	28.4%	35%	35.0%
3. Alive without mechanical ventilation	55%	64.7%	55%	62.5%
Sample size requirements				
Proportional odds model for ordinal outcome				
Expected odds ratio	–	1.50	–	1.50
Required sample size^a^	–	764	–	767
Odds ratio for binary outcome (‘Alive vs not alive’)				
Expected odds ratio^b^	–	1.50	–	4.33
Required sample size^a^	–	2526	–	326

a Total required sample size at 80% power, 5% significance level.

b Odds ratios for the binary outcome ‘Alive vs not alive’ were calculated as (0.931)(0.069)/(0.9)(0.1)=1.50 under proportional odds; and as (0.975)(0.025)/(0.9)(0.1)=4.33 under non-proportional odds.

However, there is typically no biological or statistical reason as to why the proportional odds assumption should be true, and when it is violated the interpretation of the common OR is more challenging.^9,10^ For instance, consider the data in the last two columns of Table 1. Here, the intervention has a large impact on mortality (OR for being alive 4.33), but a more modest impact on reducing the need for mechanical ventilation (OR for being alive without mechanical ventilation 1.36). The common OR is still 1.50, but this severely underestimates the intervention’s effect on mortality. Here, the common OR is calculated as some average of the two binary ORs, and thus can suffer from the same issues affecting more standard composite outcomes,^11^ where the overall treatment effect measure is not representative of the intervention’s effect on certain components of the outcome. Furthermore, instead of reducing sample size requirements, the use of an ordinal outcome in this setting actually increases the required sample size (N = 767 for ordinal outcome versus N = 326 for binary ‘Alive vs dead’ outcome).

Given the widespread use of ordinal outcomes in COVID-19 trials, we sought to empirically evaluate their use in trials for patients hospitalised with COVID-19 to determine (1) how frequently the proportional odds assumption was formally assessed and 2) how often the common OR deviated substantially compared to clinically important binary ORs constructed from the ordinal scale, including ‘Alive’, ‘Alive without mechanical ventilation’, and ‘Alive and discharged from hospital’.

## Methods

We conducted a systematic review to identify randomised trials in patients hospitalised with COVID-19, where a proportional odds model was used to analyse an ordinal clinical status outcome. For each eligible trial, we evaluated whether the proportional odds assumption was formally assessed, and then dichotomised the ordinal scale into three clinically important binary outcomes (‘Alive’, ‘Alive without mechanical ventilation’, and ‘Alive and discharged from hospital’). We then compared the OR for each of these three binary outcomes against the common OR from a proportional odds model, to assess agreement.

### Inclusion/exclusion criteria

The inclusion criteria consisted of randomised trials evaluating treatments for patients hospitalised with COVID-19, published between January 2020 and the end of May 2021, which included clinical status on an ordinal scale as a primary or secondary outcome. Studies were also required to present an overall common OR for clinical status, estimated from a proportional odds model, as well as the number of patients in each category of the ordinal scale (to facilitate calculation of binary ORs). Observational studies, literature reviews, meta-analysis, and non-randomised trials were excluded, as were studies not published in the English language. Trials that used an alternative to the proportional odds model as the main method of analysis were also excluded.

### Search strategy

MEDLINE and Embase databases were searched via OVIDSP, using the terms ‘proportional odds’ or ‘ordinal scale’, combined with ‘COVID-19’ and ‘randomised trial’. These search terms were determined by a pilot review of five COVID-19 trials.^12–16^

### Data extraction

One reviewer screened titles and abstracts to identify eligible studies. Full-text review and data extraction was then conducted independently by two reviewers, with disagreements resolved by discussion. We extracted general trial characteristics, whether the proportional odds assumptions was formally assessed (and if so, the result), the common OR estimated from a proportional odds model, and data regarding patient numbers in each category of the scale at days 7, 14–15, and 28–30, where available. These time periods were determined by pilot review, and identified to be the most frequently occurring time points at which outcomes were assessed. In trials that assessed multiple interventions, we extracted data relating to each separate comparison.

### Data analysis

#### Number of trials assessing the proportional odds assumption

Data on the number of trials where the proportional odds assumption was formally assessed (e.g. through a statistical test, such as the Brant test)^17^ were extracted and presented numerically.

#### Agreement between common and binary ORs

The ORs for the three clinically important binary outcomes (‘Alive’, ‘Alive without mechanical ventilation’, and ‘Alive and discharged from hospital’) were calculated using data on the number of patients in each category of the ordinal outcome. The ORs were standardised so that an OR > 1 indicated benefit and an OR < 1 indicated harm. Where no patients experienced an event or non-event in either the intervention group or the control group, a figure of 1 was added to each of the four categories.

In order to assess agreement between the common OR from a proportional odds model and the OR from the clinically important binary outcomes, we constructed Bland–Altman plots using log(ORs).^18^ We also evaluated the relative percentage difference of the common OR against each of the clinically relevant binary outcomes, to determine whether use of the proportional odds model was systematically over- or under-estimating the OR compared to what would be obtained using the binary outcomes. Differences were calculated on the log(OR) scale, then converted to a percentage on the OR scale through exponentiation. A percentage difference > 0, indicated the common OR was showing a larger treatment benefit compared to the OR for the binary outcome. For values < 0, this indicated the common OR was showing a smaller treatment benefit. The mean percentage difference for each outcome was calculated by weighting each comparison according to its sample size. This was to avoid allowing extreme differences between the common and binary ORs in small trials to unduly influence results. A cluster-robust variance estimator was implemented, to account for trial-level clustering.^19^ Analyses were conducted in Stata version 17.0 (StataCorp, College Station, Texas, USA).

### Sensitivity analyses

We performed sensitivity analyses to assess the robustness of the percentage difference calculations.^20^ First, we excluded analyses for which no patients experienced an event or non-event in either the intervention or control group. Second, we performed the analysis only in the set of trials, which formally assessed the proportional odds assumption and did not find any violation. Finally, we excluded analyses with extreme differences between the common OR and the binary OR, to determine whether results were being skewed by certain extreme results. We defined an extreme difference as a difference in log(ORs) of > 2 or < –2.

## Results

### Selected studies

**Figure 1.**
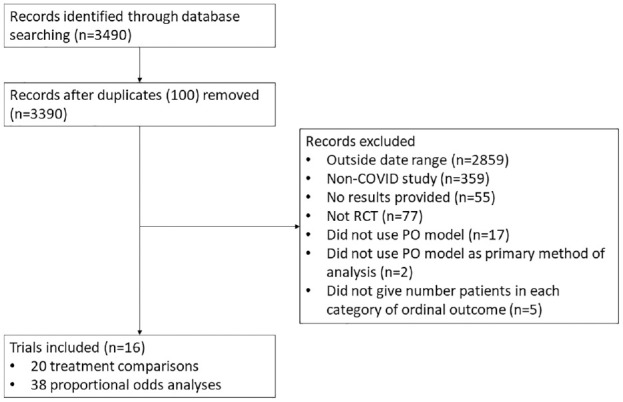
Flow diagram of study selection.

A total of 3490 records were identified through MEDLINE and Embase searches (see Figure 1), of which 16 were eligible. These 16 trials included 20 total treatment comparisons (14 trials included a single treatment comparison, and 2 trials included 3 treatment comparisons each). These 20 treatment comparisons comprised 38 total analyses (7 treatment comparisons were performed at a single time point, 8 were performed over two time points, and 5 were performed over three time points).

### Study characteristics

The characteristics of the included trials are presented in Table 2. The median (interquartile range, IQR) sample size for the treatment comparisons was 323 (265, 394), and most eligible trials used the ordinal scale as their primary endpoint (n = 10, 63%).

**Table 2.** Characteristics of included trials.

Characteristic	n/N (%)
Journal^a^	
NEJM	5/16 (31)
JAMA	4/16 (25)
Lancet	2/16 (13)
Other	5/16 (31)
Ordinal scale as primary or secondary outcome^a^	
Primary	10/16 (63)
Secondary	6/16 (38)
Number of categories of ordinal scale^a^	
6	4/16 (25)
7	8/16 (50)
8	3/16 (19)
9	1/16 (6)
Sample size per treatment comparison^b^	
Median (IQR)	323 (265, 394)
< 100	2 (10)
100–200	1 (5)
200–500	15 (75)
> 500	2 (10)
Intervention^b^	
Remdesivir	3/20 (15)
Convalescent plasma	2/20 (10)
Hydroxychloroquine	6/20 (30)
Lopinavir/ritonavir	7/20 (35)
Other	3/20 (15)
Time point of analysis^c^	
Day 7	10/38 (26)
Day 14/15	18/38 (47)
Day 28–30	10/38 (26)

IQR: interquartile range.

a Denominator is based on number of trials (N = 16).

b Denominator is based on number of treatment comparisons (N = 20).

c Denominator is based on number of analyses across all time points (N = 38).

### Proportional odds assumption

Investigators formally tested the proportional odds assumption in only 6 of 16 trials (38%), and none found evidence that the proportional odds assumption was violated.

### Agreement between common and binary ORs

**Figure 2.**
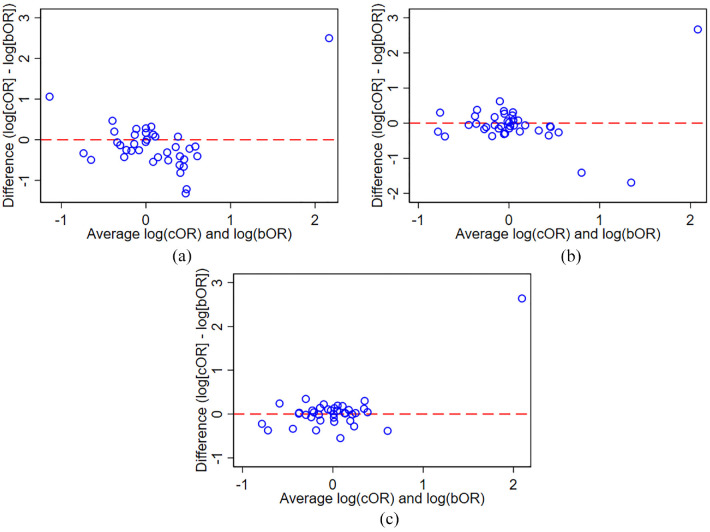
Bland–Altman plot of common OR from proportional odds model versus binary OR for three clinically important binary outcomes. (a) Outcome ‘Alive’. (b) Outcome ‘Alive without mechanical ventilation’. (c) ‘Alive and discharged from hospital’. cOR: common odds ratio; bOR: binary odds ratio.

Bland–Altman plots for the common OR from a proportional odds model versus the OR from each of the three clinically important binary outcomes are shown in Figure 2. Disagreement between the common OR from a proportional odds model and each of the binary outcomes occurred frequently: the common OR differed by more than 25% to binary OR in 21 of 38 comparisons (55%) for the outcome ‘Alive’, in 14 of 38 comparisons (37%) for the outcome ‘Alive without mechanical ventilation’, and in 9 of 38 comparisons (24%) for the outcome ‘Alive and discharged from hospital’.

The percentage difference for the common OR compared to the binary ORs is presented in Table 3. The common OR differed significantly compared to the OR for the outcome ‘Alive’ (percentage difference: –16.8%, 95% confidence interval (CI): –28.7% to –2.9%, *p* = 0.02). The estimated percentage difference of the common OR was –8.4% (95% CI: –22.6% to 8.6%, *p* = 0.29) for the outcome ‘Alive without mechanical ventilation’, and 3.6% (95% CI: –1.1% to 8.7%, *p* = 0.13) for the outcome ‘Alive and discharged from hospital’.

**Table 3.** Percentage difference of common OR from a proportional odds model against the OR of three clinically important binary outcomes.

Outcome	Mean odds ratio^a^	% difference of common OR versus binary OR^b^	*p*-value^c^
Ordinal scale	1.03	–	–
Alive	1.24	−16.8 (−28.7 to −2.9)	0.02
Alive without mechanical ventilation	1.12	−8.4 (−22.6 to 8.6)	0.29
Alive and discharged from hospital	0.99	3.6 (−1.1 to 8.7)	0.13

OR: odds ratio.

a Mean of ORs were calculated first as the mean of the log(ORs), then exponentiated.

b Percentage difference > 0 denotes the common OR is larger than the OR for the binary outcome, and thus demonstrating a larger treatment benefit. Values < 0 denote the common OR is smaller than the OR for the binary outcome.

c The *p*-value for whether percentage difference is significantly different to 0.

The common OR from a proportional odds model was statistically significant (at the 5% level) in 7 of 38 analyses (18%). In comparison, the binary OR was significant for 2 of 38 analyses (5%) for the outcome ‘Alive’, for 6 of 38 analyses (16%) for the outcome ‘Alive without mechanical ventilation’, and for 1 of 38 analyses (3%) for the outcome ‘Alive and discharged from hospital’.

### Sensitivity analyses

Results of sensitivity analyses are shown in Table S1 in the Supplementary material.

Investigators assessed and ruled out violations to the proportional odds assumption for 11 analyses across six trials. After restricting results to these analyses, the percentage difference increased for all three binary outcomes (‘Alive’–16.8% main analysis versus –27.5% sensitivity analysis; ‘Alive without mechanical ventilation’–8.4% main analysis versus –20.5% sensitivity analysis; ‘Alive and discharged from hospital’ 3.6% main analysis versus 5.9% sensitivity analysis).

Other sensitivity analyses showed similar results to the main analyses.

## Discussion

The use of the proportional odds model to analyse ordinal clinical status outcomes is common in trials of patients hospitalised with COVID-19. The proportional odds model can increase statistical efficiency and lower sample size requirements; however, the common odds ratio provided by the proportional odds model relies on the proportional odds assumption which, if violated, may lead to issues around interpretation. This review aimed to investigate how often the common OR from a proportional odds model differed to the ORs from clinically important binary outcomes in randomised clinical trials (RCTs) of COVID-19.

### Key findings

We found that a majority of trials that used the proportional odds model to estimate a common OR did not formally test the assumption underpinning this analysis. As there is generally no way to know in advance whether this assumption will hold, it is essential to properly evaluate this assumption during analysis to determine whether the interpretation of the common OR is valid. As such, in the two-thirds of trials, which did not formally test this assumption, it is difficult to ascertain the validity of the common OR as reported.

We found that the common OR from the proportional odds model often differed substantially to the OR for clinically important binary outcomes. Differences greater than 25% occurred in almost a quarter of comparisons for each of the three clinically important binary outcomes, and more than 50% of comparisons for the outcome ‘Alive’. The greater frequency of large differences for the outcome ‘Alive’ compared to the two other outcomes may be driven in part by the fact that the proportional odds model gives more weight to categories with higher number of events, and most trials will have fewer events in the ‘Dead’ category. However, given the importance of the ‘Alive vs dead’ outcome for COVID-19, this indicates that beneficial common OR from a proportional odds model should generally not be seen to denote a beneficial effect on mortality.

Interestingly, we found that even after restricting the analysis to the subset of trials, which formally tested and ruled out departures from the proportional odds assumption, the common OR still led to large differences to ORs from the three clinically important binary outcomes. This is likely explained by the relatively low power most trials have to detect violations of the proportional odds assumption.^9,17^ An alternate possibility is that even mild departures from this assumption can have large impacts on the estimated common OR. This shows that even ruling out violations to the proportional odds assumption through a formal test cannot guarantee that the proportional odds model is providing the expected interpretation.

### Interpretation

While the use of ordinal outcomes and the proportional odds model can increase efficiency and reduce sample size requirements, this requires the assumptions of proportional odds to hold; when this is not true, the proportional odds model can in fact increase sample size requirements (see Table 1). More worryingly, when the proportional odds assumption is violated, we are unaware of any clear interpretation as to what the common OR represents. It is typically interpreted as the odds of being in a higher category, yet this interpretation does not necessarily hold when the proportional odds assumption is violated; at best, the common OR can be interpreted as a weighted average of the individual binary ORs encompassing the ordinal scale, yet there is no intuitive explanation for how the categories are weighted.

To put it more succinctly, the estimand^21^ pertaining to the common OR is not well defined. Lack of clarity around the treatment effect being estimated can obscure study results, leading to inappropriate interpretations.^22,23^ For instance, as seen from our study results, a beneficial common OR does not necessarily indicate a beneficial effect on the most important categories within the ordinal outcome. This issue is similar to that of composite endpoints, where the overall effect does not necessarily represent the effect on any single component of the outcome.

One option to ensure clarity of study results is to, if feasible, use clinically relevant binary outcomes instead of ordinal outcomes. This ensures clear interpretation of what overall treatment effects represent, though it may require increased sample sizes. Notably, this is the approach taken by landmark, practice-changing trials, such as RECOVERY,^24^ which have eschewed the ordinal clinical status outcome in favour of simple binary outcomes, such as all-cause mortality.

### Limitations

The sample size for this study was relatively small (38 comparisons across 16 trials), thus leading to low statistical power when assessing differences between methods. However, this is a necessary limitation of empirical research, which by its nature is limited by the number of trials available.

Second, the main aim of this study was to assess agreement between common and binary ORs. As such, we only included studies that used a proportional odds model to estimate a common OR. Therefore, our results pertaining to the number of trials that formally assessed the proportional odds assumption excludes trials, which planned to use a proportional odds model but changed to an alternative method of analysis after finding violations to this assumption. Of note, two trials were excluded because they did not use the proportional odds model after finding violations to the proportional odds assumption; including these trials would change our results so that 8/18 (44%) trials assessed the proportional odds assumption, with 2/8 (25%) finding statistical evidence against the assumption.

Third, as discussed above, the observed differences between the common odds ratio and the odds ratio for the outcome ‘Alive’ may be in part driven by the small number of events in the ‘Dead’ category. This could be because the included trials enrolled lower-risk populations who were less likely to die. Had our sample been made up of trials with higher-risk populations, with higher mortality rates, it is possible we would have observed better agreement between the two methods. This highlights that disagreement between the common odds ratio and odds ratios from binary outcomes may be driven by numerous factors, including not only whether the proportional odds assumption is violated, but also the type of population enrolled in the study and the number of events in each category of the ordinal scale.

Fourth, although we have accounted for random variability in comparison of the percentage difference between the common versus binary odds ratios, we have not done so in our summary of the proportion of trials with a greater than 25% difference between the two methods. Thus, we cannot say to what extent these results may be driven by chance.

Finally, this article has focussed on treatment effect estimation; however, there is a distinction between estimation and hypothesis testing. Importantly, it has been shown that the score test from a proportional odds model is asymptotically equivalent to the Wilcoxon–Mann–Whitney test, which measures the probability that a randomly selected intervention-arm participant will have a better outcome than a randomly selected control-arm participant.^25^ Thus, even when the proportional odds assumption is not true, the proportional odds model still provides a valid test of the null hypothesis.

## Conclusion

The common OR from proportional odds models often differs substantially to ORs from clinically important binary outcomes, and, similar to composite outcomes, a beneficial common OR from a proportional odds model does not necessarily indicate a beneficial effect on the most important categories within the ordinal outcome.

## Supplemental Material

sj-docx-1-ctj-10.1177_17407745231211272 – Supplemental material for Evaluating whether the proportional odds models to analyse ordinal outcomes in COVID-19 clinical trials is providing clinically interpretable treatment effects: A systematic review Supplemental material, sj-docx-1-ctj-10.1177_17407745231211272 for Evaluating whether the proportional odds models to analyse ordinal outcomes in COVID-19 clinical trials is providing clinically interpretable treatment effects: A systematic review by Masuma Uddin, Nasir Z Bashir and Brennan C Kahan in Clinical Trials
