# Supplementary material for: Evaluating whether the proportional odds models to analyse ordinal outcomes in COVID-19 clinical trials is providing clinically interpretable treatment effects: A systematic review
Source: Clin Trials. 2023 Nov 20;21(3):363–70. doi: 10.1177/17407745231211272 (PMC11134983; doi:10.1177/17407745231211272)
Supplement: sj-docx-1-ctj-10.1177_17407745231211272 – Supplemental material for Evaluating whether the proportional odds models to analyse ordinal outcomes in COVID-19 clinical trials is providing clinically interpretable treatment effects: A systematic review [file sj-docx-1-ctj-10.1177_17407745231211272.docx]

**Supplementary material: *Evaluating whether the proportional odds models to analyse ordinal outcomes in COVID-19 clinical trials is providing clinically interpretable treatment effects: A systematic review***

**List of included articles**

1. A Neutralizing Monoclonal Antibody for Hospitalized Patients with Covid-19. New England Journal of Medicine. 2020;384(10):905-14.

2. Ader F, Peiffer-Smadja N, Poissy J, Bouscambert-Duchamp M, Belhadi D, Diallo A, et al. An open-label randomized controlled trial of the effect of lopinavir/ritonavir, lopinavir/ritonavir plus IFN-β-1a and hydroxychloroquine in hospitalized patients with COVID-19. Clin Microbiol Infect. 2021;27(12):1826-37.

3. Beigel JH, Tomashek KM, Dodd LE, Mehta AK, Zingman BS, Kalil AC, et al. Remdesivir for the Treatment of Covid-19 — Final Report. New England Journal of Medicine. 2020;383(19):1813-26.

4. Cavalcanti AB, Zampieri FG, Rosa RG, Azevedo LCP, Veiga VC, Avezum A, et al. Hydroxychloroquine with or without Azithromycin in Mild-to-Moderate Covid-19. New England Journal of Medicine. 2020;383(21):2041-52.

5. Furtado RHM, Berwanger O, Fonseca HA, Corrêa TD, Ferraz LR, Lapa MG, et al. Azithromycin in addition to standard of care versus standard of care alone in the treatment of patients admitted to the hospital with severe COVID-19 in Brazil (COALITION II): a randomised clinical trial. The Lancet. 2020;396(10256):959-67.

6. Kalil AC, Patterson TF, Mehta AK, Tomashek KM, Wolfe CR, Ghazaryan V, et al. Baricitinib plus Remdesivir for Hospitalized Adults with Covid-19. New England Journal of Medicine. 2020;384(9):795-807.

7. Liesenborghs L, Spriet I, Jochmans D, Belmans A, Gyselinck I, Teuwen LA, et al. Itraconazole for COVID-19: preclinical studies and a proof-of-concept randomized clinical trial. EBioMedicine. 2021;66:103288.

8. López-Medina E, López P, Hurtado IC, Dávalos DM, Ramirez O, Martínez E, et al. Effect of Ivermectin on Time to Resolution of Symptoms Among Adults With Mild COVID-19: A Randomized Clinical Trial. JAMA. 2021;325(14):1426-35.

9. O’Donnell MR, Grinsztejn B, Cummings MJ, Justman JE, Lamb MR, Eckhardt CM, et al. A randomized double-blind controlled trial of convalescent plasma in adults with severe COVID-19. The Journal of Clinical Investigation. 2021;131(13).

10. Réa-Neto Á, Bernardelli RS, Câmara BMD, Reese FB, Queiroga MVO, Oliveira MC. An open-label randomized controlled trial evaluating the efficacy of chloroquine/hydroxychloroquine in severe COVID-19 patients. Scientific Reports. 2021;11(1):9023.

11. Sehgal IS, Guleria R, Singh S, Siddiqui MS, Agarwal R. A randomised trial of Mycobacterium w in critically ill patients with COVID-19: ARMY-1. ERJ Open Res. 2021;7(2).

12. Self WH, Semler MW, Leither LM, Casey JD, Angus DC, Brower RG, et al. Effect of Hydroxychloroquine on Clinical Status at 14 Days in Hospitalized Patients With COVID-19: A Randomized Clinical Trial. JAMA. 2020;324(21):2165-76.

13. Simonovich VA, Burgos Pratx LD, Scibona P, Beruto MV, Vallone MG, Vázquez C, et al. A Randomized Trial of Convalescent Plasma in Covid-19 Severe Pneumonia. New England Journal of Medicine. 2020;384(7):619-29.

14. Spinner CD, Gottlieb RL, Criner GJ, Arribas López JR, Cattelan AM, Soriano Viladomiu A, et al. Effect of Remdesivir vs Standard Care on Clinical Status at 11 Days in Patients With Moderate COVID-19: A Randomized Clinical Trial. JAMA. 2020;324(11):1048-57.

15. Tomazini BM, Maia IS, Cavalcanti AB, Berwanger O, Rosa RG, Veiga VC, et al. Effect of Dexamethasone on Days Alive and Ventilator-Free in Patients With Moderate or Severe Acute Respiratory Distress Syndrome and COVID-19: The CoDEX Randomized Clinical Trial. JAMA. 2020;324(13):1307-16.

16. Wang Y, Zhang D, Du G, Du R, Zhao J, Jin Y, et al. Remdesivir in adults with severe COVID-19: a randomised, double-blind, placebo-controlled, multicentre trial. The Lancet. 2020;395(10236):1569-78.

**Table S1 – Sensitivity analyses for percentage difference results**

| **Outcome** | **Percentage difference of common OR vs. binary OR** | **P-value** |
| --- | --- | --- |
| ***Main analysis*** |  |  |
| Alive**^a^** | -16.8 (-28.7 to -2.9) | 0.02 |
| Alive without mechanical ventilation**^a^** | -8.4 (-22.6 to 8.6) | 0.29 |
| Alive and discharged from hospital**^a^** | 3.6 (-1.1 to 8.7) | 0.13 |
| ***Excluding comparisons with extreme differences^b^*** |  |  |
| Alive^c^ | -19.1% (-32.0% to -3.7%) | 0.02 |
| Alive without mechanical ventilation^c^ | -6.7 (-21.2% to 10.5%) | 0.40 |
| Alive and discharged from hospital^c^ | 4.9% (-1.7% to 12.1%) | 0.14 |
| ***Including only trials where proportional odds assumption was assessed and not violated*** |  |  |
| Alive^d^ | -33.9% (-53.0% to -7.1%) | 0.03 |
| Alive without mechanical ventilation^d^ | -11.9% (-49.0% to 51.9%) | 0.58 |
| Alive and discharged from hospital^d^ | 14.3% (-5.4% to 38.0%) | 0.13 |
| ***Excluding comparisons with no events in any cells*** |  |  |
| Alive^e^ | -17.9% (-32.4% to -0.4%) | 0.03 |
| Alive without mechanical ventilation^c^ | -1.5% (-13.5% to 12.3%) | 0.82 |
| Alive and discharged from hospital^f^ | NA | NA |

^a^ N=38 comparisons included in analysis

^b^ Differences between the binary OR and common OR were defined as extreme if log(cOR) – log(bOR) was greater than 2 or less than -2

^c^ N=37 comparisons included in analysis

^d^ N=11 comparisons included in analysis

^e^ N=34 comparisons included in analysis

^f^ All comparisons had ≥1 event in each cell

**Table S2 – Results for common and binary odds ratios for each comparison**

| Trial number | Treatment comparison number | Time point | Sample size for comparison | Common OR | Binary OR for "Alive" | Binary OR for "Alive without mechanical ventilation" | Binary OR for "Alive and discharged home" |
| --- | --- | --- | --- | --- | --- | --- | --- |
| 1 | 1 | 15 | 391 | 1.65 | 1.95 | 8.95 | 1.23 |
| 2 | 2 | 28 | 223 | 1.50 | 2.25 | 1.65 | 1.33 |
| 3 | 3 | 15 | 1062 | 1.50 | 1.87 | 1.67 | 1.44 |
| 4 | 4 | 7 | 314 | 0.85 | 0.53 | 0.58 | 0.86 |
| 5 | 5 | 15 | 42 | 30.40 | 2.50 | 2.11 | 2.17 |
| 5 | 6 | 28 | 42 | 1.10 | 1.50 | 4.50 | 1.46 |
| 6 | 7 | 15 | 105 | 0.41 | 0.67 | 0.60 | 0.51 |
| 6 | 8 | 28 | 105 | 0.40 | 0.56 | 0.52 | 0.59 |
| 6 | 9 | 7 | 105 | 0.54 | 0.19 | 0.40 | 0.76 |
| 7 | 10 | 15 | 1033 | 1.30 | 1.55 | 1.85 | 1.27 |
| 8 | 11 | 15 | 397 | 0.74 | 0.96 | 0.82 | 0.75 |
| 8 | 12 | 28 | 397 | 0.70 | 0.90 | 0.83 | 0.68 |
| 8 | 13 | 7 | 397 | 0.63 | 0.96 | 0.66 | 0.49 |
| 9 | 14 | 15 | 331 | 1.01 | 1.68 | 0.99 | 0.81 |
| 9 | 15 | 7 | 331 | 1.23 | 2.00 | 0.66 | 1.25 |
| 9 | 16 | 15 | 332 | 0.83 | 0.92 | 1.11 | 0.80 |
| 9 | 17 | 7 | 332 | 1.09 | 0.92 | 0.83 | 0.95 |
| 9 | 18 | 15 | 331 | 1.22 | 1.83 | 0.89 | 1.02 |
| 9 | 19 | 7 | 331 | 1.12 | 2.18 | 0.80 | 1.31 |
| 10 | 20 | 15 | 299 | 1.52 | 1.41 | 1.97 | 2.22 |
| 11 | 21 | 15 | 400 | 1.09 | 2.03 | 1.01 | 1.01 |
| 11 | 22 | 7 | 400 | 1.01 | 1.01 | 0.96 | 1.01 |
| 12 | 23 | 15 | 65 | 0.83 | 3.09 | 0.97 | 1.43 |
| 13 | 24 | 15 | 479 | 1.02 | 0.78 | 1.09 | 0.94 |
| 13 | 25 | 28 | 479 | 0.97 | 1.02 | 1.06 | 1.05 |
| 13 | 26 | 7 | 479 | 1.16 | 1.02 | 1.23 | 0.96 |
| 14 | 27 | 15 | 333 | 1.00 | 2.26 | 1.27 | 0.90 |
| 14 | 28 | 28 | 333 | 0.81 | 1.05 | 1.11 | 0.94 |
| 14 | 29 | 7 | 333 | 0.88 | 2.97 | 0.96 | 0.62 |
| 15 | 30 | 15 | 236 | 1.25 | 0.91 | 1.54 | 1.14 |
| 15 | 31 | 28 | 236 | 1.15 | 0.87 | 1.07 | 1.13 |
| 15 | 32 | 7 | 236 | 0.69 | 0.79 | 1.00 | 1.00 |
| 16 | 33 | 15 | 293 | 0.83 | 1.43 | 0.88 | 0.77 |
| 16 | 34 | 28 | 293 | 0.93 | 0.83 | 0.78 | 1.11 |
| 16 | 35 | 15 | 293 | 0.69 | 0.74 | 0.70 | 0.68 |
| 16 | 36 | 28 | 293 | 0.76 | 0.62 | 0.62 | 0.82 |
| 16 | 37 | 15 | 293 | 0.93 | 1.43 | 1.08 | 0.81 |
| 16 | 38 | 28 | 293 | 1.16 | 1.07 | 0.93 | 1.14 |
